# The X-ray Sensitivity of an Amorphous Lead Oxide Photoconductor

**DOI:** 10.3390/s21217321

**Published:** 2021-11-03

**Authors:** Oleksandr Grynko, Tristen Thibault, Emma Pineau, Alla Reznik

**Affiliations:** 1 Chemistry and Materials Science Program, Lakehead University, Thunder Bay, ON P7B 5E1, Canada; 2 Physics Department, Lakehead University, Thunder Bay, ON P7B 5E1, Canada; ttthibau@lakeheadu.ca (T.T.); enpineau@lakeheadu.ca (E.P.); areznik@lakeheadu.ca (A.R.); 3 Thunder Bay Regional Health Research Institute, Thunder Bay, ON P7B 6V4, Canada

**Keywords:** lead oxide, X-ray detector, direct conversion, X-ray sensitivity, columnar recombination, Langevin recombination, Monte Carlo simulation

## Abstract

The photoconductor layer is an important component of direct conversion flat panel X-ray imagers (FPXI); thus, it should be carefully selected to meet the requirements for the X-ray imaging detector, and its properties should be clearly understood to develop the most optimal detector design. Currently, amorphous selenium (a-Se) is the only photoconductor utilized in commercial direct conversion FPXIs for low-energy mammographic imaging, but it is not practically feasible for higher-energy diagnostic imaging. Amorphous lead oxide (a-PbO) photoconductor is considered as a replacement to a-Se in radiography, fluoroscopy, and tomosynthesis applications. In this work, we investigated the X-ray sensitivity of a-PbO, one of the most important parameters for X-ray photoconductors, and examined the underlying mechanisms responsible for charge generation and recombination. The X-ray sensitivity in terms of electron–hole pair creation energy, *W_±_*, was measured in a range of electric fields, X-ray energies, and exposure levels. *W_±_* decreases with the electric field and X-ray energy, saturating at 18–31 eV/ehp, depending on the energy of X-rays, but increases with the exposure rate. The peculiar dependencies of *W_±_* on these parameters lead to a conclusion that, at electric fields relevant to detector operation (~10 V/μm), the columnar recombination and the bulk recombination mechanisms interplay in the a-PbO photoconductor.

## 1. Introduction

The ever-growing demand for advanced radiation medical imaging techniques sustains continued research interest in novel materials and technologies for imaging detectors, based on the direct conversion of diagnostic X-rays. In direct conversion flat panel X-ray imagers (FPXIs), a uniform layer of the photoconductor is deposited over large area readout electronics based on either thin-film transistor (TFT) arrays or complementary metal-oxide-semiconductor (CMOS) active-matrix arrays. The photoconductor acts as a direct X-ray-to-charge transducer; i.e., it absorbs X-rays and directly creates electron–hole pairs (ehps), which are subsequently separated by a bias field to generate a signal.

Stabilized amorphous selenium (a-Se) is the most successful, commercially viable, large-area-compatible X-ray photoconductor used in direct conversion FPXIs for medical imaging due to its several distinct advantages over other potentially competing photoconductors [1,2]. Both X-ray-generated electrons and holes can drift in a-Se under appropriate conditions [3,4]. The dark current can be appropriately controlled by the use of blocking structures [5,6]. The X-ray attenuation coefficient, while not outstanding, is acceptable for the relatively soft X-rays in mammographic energy range (~20 keV) [1,3]. The fabrication technology of the practical photoconductive layers is mature enough, and thus cost-effective. Therefore, the most successful application of stabilized a-Se technology is in mammography where a-Se-based FPXIs became a dominant technology [1,7]. However, for higher energy applications such as radiography, fluoroscopy, and tomosynthesis, a-Se does not have sufficient X-ray stopping power, and thus, alternative materials are needed to expand the success of the direct conversion concept over the diagnostic energy range. In this regard, one should focus on the development of high-*Z* (atomic number), wide-bandgap photoconductors that can efficiently attenuate high-energy X-ray photons and are compatible with the FPXI technology. The latter requires the feasibility to be deposited over the imaging array under deposition conditions suitable for imaging electronics, such as a deposition temperature that imaging electronics can withstand (practically below 250 °C), a high deposition rate, and high uniformity across a large area [1].

The requirement for a relatively low deposition temperature makes single-crystalline photoconductors unsuitable for use in direct conversion imaging detectors. As for the disordered (polycrystalline and amorphous), high-*Z* semiconductors that can produce large-area detectors, polycrystalline layers of PbI_2_ [8,9,10], HgI_2_ [10,11,12], CdTe [13], Cd_1-x_Zn_x_Te [14], BiI_3_ [15], ZnO [16], PbO [17,18], perovskites [19], and amorphous PbO (a-PbO) [20,21] are considered promising. However, at the current stage of their technology, the majority of the materials in this list exhibit signal lag—a residual current that continues to flow after X-ray exposure [17,18,22]. The presence of this residual signal has a detrimental effect on procedures with fast sequential image acquisition, such as real-time imaging (fluoroscopy) and 3D imaging (tomosynthesis), since part of the signal from a previous exposure combines with the next one. The resulting image can be inaccurate, misleading, and as such, can compromise the whole visualization advantage of real-time (fluoroscopic) imaging. For practical fluoroscopic applications, the residual signal should promptly decay to <10% in less than 33 ms, i.e., within the first frame, appertaining to a 30 frames per second (fps) read-out [23,24,25,26,27]. To the best of our knowledge, today, the only photoconductors exempted from the issue of signal lag are a-Se [5,28] and amorphous lead oxide—a new non-crystalline polymorph of PbO [29], in which signal lag was suppressed to a level <5% [20] that fits the requirements of fluoroscopic applications. In the continuous advancement of a-PbO technology, we recently reported on the development of a blocking structure for a-PbO detectors, in which a thin layer of polyimide (PI) is introduced between the electrode and a-PbO layer, thereby preventing their interaction. This blocking structure is needed to maintain low dark current (<1 pA/mm^2^) in a strong operational electric field (≥10 V/μm), while preserving the temporal performance suitable for real-time imaging with lag values down to 0.9% at 30 fps read-out [21].

One of the most important parameters for X-ray photoconductors is its X-ray sensitivity, characterized in terms of the ehp creation energy $W_{\pm }$, which is the average energy required to generate a single detectable electron and hole pair. The lower the $W_{\pm }$, the higher the X-ray sensitivity of a photoconductor. This parameter accounts solely for the freed carriers, reflecting the fact that only a fraction of the X-ray-generated charges are collected. Indeed, a theoretical (or, intrinsic) ehp creation energy, $W_{\pm }^{0}$, can be estimated from the bandgap of the semiconductor, $E_{g}$, by an empirical formula, the so-called Klein rule: $W_{\pm }^{0}\approx 3E_{g}$ [30]. However, in practice, in many photoconductors, including a-Se [31], poly-PbO [17,18,22], and a-PbO [20] ones, experimentally measured (or, effective) $W_{\pm }$ is not a material parameter, but a characteristic of the system which depends on the applied electric field *F*, X-ray energy *E,* photon flux (or exposure *X*), and temperature *T*. Understanding of $W_{\pm }\left(F,E,X,T\right)$ dependencies in a-PbO photoconductors is crucial for the development and optimization of an a-PbO-based direct conversion X-ray imaging detector.

In this work, we investigated the X-ray sensitivity of an a-PbO photoconductor in terms of $W_{\pm }$ and examined the underlying mechanisms responsible for charge generation and recombination in this material through experimental measurements of $W_{\pm }$ and Monte Carlo simulations of photoelectron transport. $W_{\pm }$ was measured in a range of electric fields, X-ray energies, and exposures. The peculiar dependency of X-ray sensitivity on these parameters leads to a conclusion that the interplay of the columnar and bulk recombination mechanisms dominates in the a-PbO photoconductor at electric fields relevant to detector operation (i.e., 10 V/μm). Finally, we suggest a qualitative model for the charge generation and recombination processes in the a-PbO photoconductor that is supported by a Monte Carlo simulation.

## 2. Background

It was suggested that the intrinsic ehp creation energy, $W_{\pm }^{0}$, of a semiconductor depends on its bandgap $E_{g}$ according to the relationship $W_{\pm }^{0}\approx 2.8E_{g}+\varepsilon_{ph}$ (Klein rule for crystalline semiconductors [30]) or $W_{\pm }^{0}\approx 2.2E_{g}+\varepsilon_{ph}$ (Que–Rowlands rule for amorphous solids [32]), where the term $\varepsilon_{ph}\approx 0.5-1\;\mathrm{eV}$ is responsible for losses due to optical phonons. In practice, many low-mobility amorphous and polycrystalline semiconductors demonstrate effective $W_{\pm }$ that is higher than the intrinsic value. For example, $W_{\pm }$ is ~45 eV/ehp for a-Se [3], ~17 eV/ehp for poly-PbO [18], and ~22 eV/ehp for a-PbO [20] at a practical electric field of *F* = 10 V/μm, whereas theoretical values are within 5–7 eV/ehp. The fact that experimental $W_{\pm }$ exceeds the theoretical value indicates that a certain portion of the initially X-ray-generated charge undergoes deep trapping or recombination and thus does not contribute to the photo-signal, reducing the detector’s sensitivity, and ultimately degrading the SNR of the image.

Generally speaking, the carriers can be trapped at localized states within the mobility gap of a-PbO, in either shallow or deep traps. However, a previous investigation of the ghosting effect [21] suggested that no deep trapping occurs in PI/a-PbO photoconductive structures, at least at the relatively low exposures used in this study. Ghosting is caused by deep bulk trapping of photogenerated carriers, which subsequently recombine with the drifting carriers of the opposite sign, resulting in sensitivity degradation. Since no detectable ghosting effect was observed at relevant exposure rates, deep trapping can be ruled out as a primary cause for $W_{\pm }$ degradation. Additionally, the quasi-rectangular shape of the X-ray response indicates the unrestricted flow of the photogenerated carriers from the a-PbO photoconductive layer through the PI blocking layer into the ITO electrode [21], meaning that no accumulation of carriers in shallow states at the PI/a-PbO interface are expected as well. Therefore, a trapping mechanism can be excluded from the reasons for the carrier loss in a-PbO and will not be discussed further.

As for the recombination, there are three main theories that could explain the loss of the X-ray-generated carriers in the photoconductors: the bulk (Langevin), geminate (Onsager), and columnar (track) models [32,33,34,35,36,37,38,39,40,41]. Bulk Langevin recombination is a bimolecular process in which electrons and holes drift through the bulk of the photoconductor, due to the internal electric field within the layer, meet each other in space and time, and recombine. The two other intra-track mechanisms, i.e., geminate and columnar recombination, occur within the ionization column formed along the track of the energetic primary photoelectron. In the geminate model described by Onsager theory [42], the twin generated electron and hole pair recombine with each other while diffusing and drifting in the presence of their mutual Coulomb attraction and the applied electric field. Columnar recombination, first proposed by Jaffe [43] and expanded by Hirsch and Jahankhani [44], assumes that the photogenerated charge density inside the column is high enough so that the concept of independent geminate ehps is inapplicable. In this case, bimolecular recombination occurs between two non-geminate charges (i.e., electron and hole from two different twin pairs), just like in the bulk Langevin model, but within the ionization column.

The applicability of the recombination models depends on the properties of the material under consideration, and also on the source of excitation. For example, it was shown that the recombination of drifting holes with either drifting or trapped electrons in a-Se follows the bulk Langevin recombination mechanism [45,46]; initial recombination of optically excited carriers is controlled by the geminate mechanism [47], but columnar recombination prevails in the case of X-ray photogeneration [2,33,34,36]. On the other hand, geminate recombination controls the effective $W_{\pm }$ in X-ray irradiated anthracene, PVK, and in electron-bombarded SiO_2_ ([32,39,40] and references therein). Although these materials have some common properties (i.e., low mobility), they have different recombination mechanisms. Therefore, one cannot rule out any of these theories a priori, but must first assess their fitness based on the experimental results. Conveniently, the recombination rate of each mechanism depends uniquely on experimental parameters such as electric field, exposure, X-ray photon energy, and temperature, which can be used to identify the dominant process.

### 2.1. Exposure Dependency

Bulk bimolecular recombination in amorphous solids is usually described using the Langevin formalism, which states that the recombination rate is proportional to the concentration of both types of carriers. Therefore, if bulk recombination is a dominant process, the collected charge *Q* should change with exposure X according to $Q\sim X^{1/2}$ [40].

The situation is different for the intra-track mechanisms. With increasing radiation intensity, the number of primary photoelectron tracks proportionally increases, but the recombination within each track remains unaffected. This means that for geminate and columnar mechanisms, the collected charge increases linearly with the exposure, following Q~X [39]. Additionally, geminate recombination is a monomolecular process; therefore, the recombination probability does not depend on the concentration of the surrounding charges (since the separation between the geminate electron and hole is the smallest distance between any two oppositely charged carriers), and thus the relationship Q~X is adhered to again [41].

### 2.2. Field Dependency

The X-ray sensitivity in many X-ray photoconductors (i.e., a-Se, poly-PbO, a-PbO, perovskites) shows a very pronounced electric field dependency [3,18,20,48]. It is usually described as $W_{\pm }\left(F\right)=W_{\pm }^{0}+B/F$, where $W_{\pm }^{0}$ is the intrinsic ehp creation energy at an “infinite” field and B is a material-specific constant that depends on the energy of X-ray photons ([1,33] and references therein).

Regardless of the mechanism, the recombination rate is determined by the probability of carriers meeting in space. It ultimately depends on the interplay between three main driving forces: charge carrier thermal diffusion, charge carrier drift in the applied electric field, and mutual attraction between the oppositely charged carriers. The applied electric field acts to overcome mutual Coulombic attraction between photogenerated electrons and holes, increasing the recombination escape probability. This results in a higher number of freed ehps and lower $W_{\pm }$ [36].

Such field-dependent sensitivity is typical for both columnar and geminate recombination ([33,34] and references therein), although each of them has its own peculiarities. In the columnar model, at the very low electric fields (≲1 V/μm, when diffusion dominates over drift), $W_{\pm }$ is field-independent [41]. In the geminate model, the low-field portion of the photogeneration efficiency $\eta \left(F\right)=W_{\pm }^{0}/W_{\pm }\left(F\right)$ is a straight line with a slope-to-intercept ratio $R_{SI}=e^{3}/\left(8\pi \varepsilon_{r}\varepsilon_{0}k^{2}T^{2}\right)$, where e—elementary charge, $\varepsilon_{r}$—relative permittivity of the photoconductor, $\varepsilon_{0}$—vacuum permittivity, and k—Boltzmann’s constant ([33] and references therein).

The fraction of carriers lost to bulk recombination is proportional to $F^{-2}$, and thus the collected charge is given by $Q\sim 1/\left(1+F^{-2}\right)$ [41,49].

### 2.3. X-ray Energy Dependency

To the best of our knowledge, the only photoconductor whose X-ray energy dependency on $W_{\pm }$ has been examined (both experimentally [31,41,50,51,52] and theoretically [34,35,37,53,54,55]) is a-Se. As was discussed in [34] (and references therein), within the framework of the geminate recombination model, the initial separation between an electron and a hole controls the probability of their escape from recombination. Therefore, if the initial separation is independent of the incident photon energy, $W_{\pm }$ should be too, if the geminate recombination is the dominant process.

On the other hand, through the example of a-Se, it has been shown that the columnar recombination rate drops with increasing X-ray photon energy [33,34,53]. This is due to a rise in the mean separation of the electrons and holes within the ionization column. As the charge density decreases, the recombination rate between non-geminate electrons and holes within the column also declines. This increases the number of free electrons and holes which, in turn, leads to a reduction in $W_{\pm }$.

## 3. Materials and Methods

### 3.1. Detector Preparation

A single-pixel direct conversion digital detector based on an amorphous lead oxide (a-PbO) photoconductor with a single blocking layer of polyimide (PI) was used in this work. Commercially supplied pre-washed and vacuum-packed ITO-coated glass (bottom biasing electrode) was rinsed with acetone, methanol, and isopropanol; dried with dry nitrogen; and placed on a hot plate at 90 °C for 10 min, to ensure cleanliness. A 1 μm thick PI layer was then spin-coated onto the ITO-coated glass. 19 μm of a-PbO was deposited on the prepared substrate by ion-assisted thermal evaporation. Finally, a top Au contact (readout electrode) 1.1 mm in diameter was sputtered atop of the a-PbO, which provided an effective detector area of 0.95 mm^2^. Detailed descriptions of the PI application and a-PbO deposition can be found in [21,56].

The density of the a-PbO photoconductor *ρ* was calculated from the mass *m* and volume of the film, which can be treated as a cylinder with a height *d* and radius *r*; thus, $\rho =m/\left(\pi r^{2}d\right)$. The a-PbO deposition was performed using a shadow mask with a window of radius *r* = 6.25 mm. The photoconductor thickness *d* = 19 μm was measured with a stylus profilometer (KLA Tenchor Alpha-Step D-100, Milpitas, CA, USA). The glass substrate with the applied PI layer was weighed on a microbalance (Sartorius CP2P, Göttingen, Germany) before and after deposition of a-PbO film to calculate the mass of the photoconductor layer: *m* = 20.5 mg. The density was found to be *ρ* = 8.8 g/cm^3^, which was 92% of the crystalline PbO density (9.53 g/cm^3^), owing to high packing density and the absence of voids in the a-PbO layer [56].

### 3.2. Experimental Apparatus

**Figure 1:**
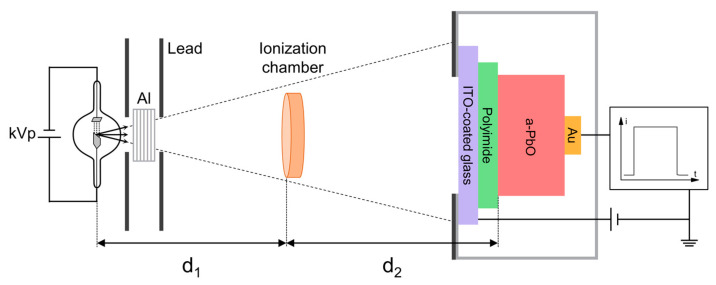
Schematics of the XPM setup (not to scale).

X-ray characterization of the PI/a-PbO detector was performed using the X-ray-induced photocurrent method (XPM). The experimental setup is shown in Figure 1. A detector was placed in a shielded aluminum box. Prior to measurement, the detector was short-circuited in the dark to allow for the complete detrapping of charge carriers. A positive dc bias was applied to the ITO by a high voltage power supply (Stanford Research Systems PS350, Sunnyvale, CA, USA) to create a strong electric field in the photoconductor. The photocurrent due to the drifting carriers was read out from the Au electrode by an oscilloscope (Tektronix TDS 2024C, Beaverton, OR, USA) with a native input resistance of 1 MΩ. In this work, the electric field is referred to as an applied field to the detector $F=V_{bias}/\left(d_{PbO}+d_{PI}\right)$, where $V_{bias}$ is an applied bias, and $d_{PbO}$ and $d_{PI}$ are the thicknesses of the a-PbO and PI layers, respectively. After such a bias is applied, the dark current in the PI/a-PbO detector exponentially decreases with time due to the accumulation of trapped charge within the PI blocking layer [21]. Therefore, the bias was applied to the detector for 15 min prior to irradiation to allow the dark current to stabilize and to drop to a level below 5 pA/mm^2^. An X-ray unit (tube Dunlee PX1412CS, insert DU-304, generator CPI Indico 100, Georgetown, ON, Canada) with a tungsten target was used to generate X-ray pulses. The tube voltage could be varied in the range of 40–100 kVp and the tube current could be set in between 25 mA and 400 mA. 2-mm Lead collimators were used to form a narrow-beam geometry and to minimize scattering. An added filtration of Aluminum (type 1100, min 99.0% purity) was placed in the cassette in front of the X-ray tube to harden the X-ray beam. The exposure was monitored by dosimeter Keithley 35040 (Cleveland, OH, USA) with ionization chamber Keithley 96035 (Cleveland, OH, USA). The ion chamber was positioned midway between the detector and the tube to avoid any contribution of backscattered X-rays to the exposure reading.

$W_{\pm }$ is derived as a ratio of the total energy absorbed in the photoconductor upon X-ray irradiation $E_{abs}$ to the number of collected ehps $N_{ehp}$:

(1) $$W_{\pm }=\frac{E_{abs}}{N_{ehp}}\;.$$

A detailed description of calculation of the absorbed energy, the number of collected charges, and X-ray sensitivity is provided in Appendix A.

### 3.3. Monte Carlo Simulations

**Figure A1:**
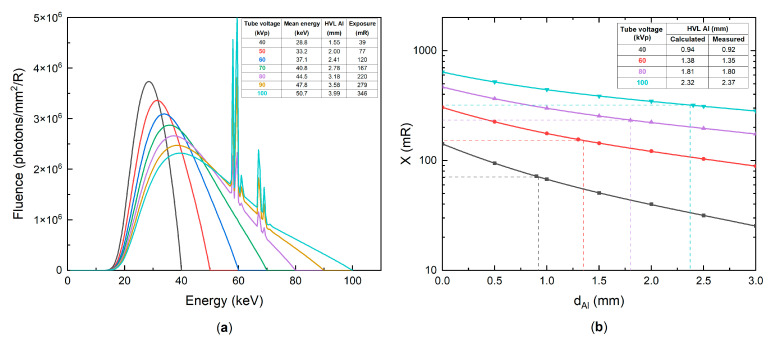
(**a**) The simulated X-ray spectra at different tube voltages, normalized to 1 R of exposure. The inset shows typical (see text) parameters of the beams. (**b**) Exposure measured as a function of added Al filtration thickness for different tube voltages. The dashed lines correspond to 50% of the original exposure and HVL of Al. The inset shows calculated and measured HVL values for a naked tube.

Monte Carlo simulations of the electron trajectories in PbO were performed using the Stopping and Range of Electrons in Matter (SREM)-type Monte Carlo software CASINO (monte CArlo SImulation of electroNs in sOlids) [57]. A PbO sample was irradiated with an electron beam and transport of electrons was simulated, taking into account the physical interaction with the matter. The electron beam energies were selected to represent the kinetic energy of the ejected primary photoelectrons KE=hν−BE, where hν is the mean energy of the incident X-ray photons in the beam and BE is the binding energy of that photoelectron. Since the mean energies of the X-ray beams used in this work (see Figure A1a) were lower than the *K*-edge energy of PbO ($BE_{K}=88\;\mathrm{keV}$), the photoelectrons were considered to be ejected from the *L*_3_-subshell with binding energy $BE_{L_{3}}=13\;\mathrm{keV}$ [58].

The Monte Carlo simulation method and the physical models used were described in [59,60]. For each beam energy, the trajectory information (such as collision event coordinates and energy) from the 500 primary electrons, which included ~10^5^–10^6^ events (depending on the beam energy), was recorded and further analyzed. The energy difference between two consecutive events was calculated and used as the dissipated energy per collision event, and the coordinates were used to calculate a distance between these consecutive collision sites and the average total path length. Finally, the average ratio of the dissipated energy to the distance between collision sites was calculated for each electron beam energy, which can be treated as the rate of energy deposited in the photoconductor.

## 4. Results

**Figure 2:**
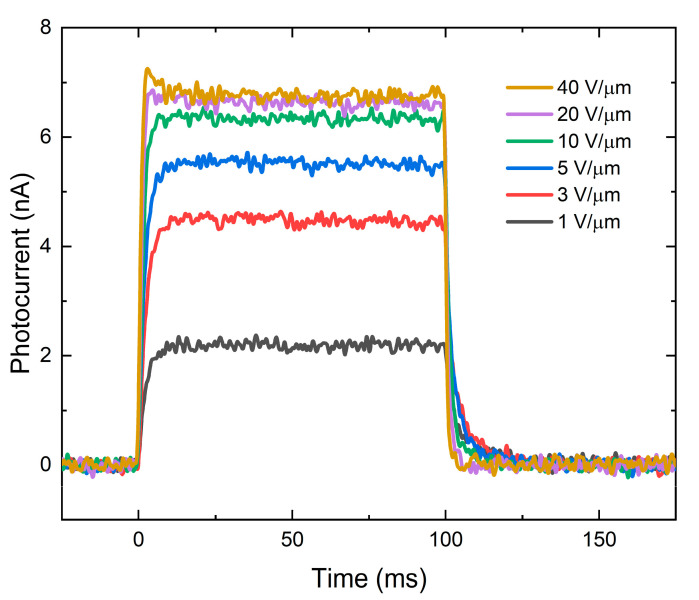
A typical X-ray response to 60 kVp irradiation at different electric fields.

Figure 2 shows a typical X-ray response of the PI/a-PbO detector to irradiation by a 100-ms X-ray pulse at different applied electric fields and a tube voltage of 60 kVp. Without irradiation, the detector produces only dark current in the order of several picoamps. Upon X-ray irradiation, the detector exhibits a quasi-rectangular response with a uniform amplitude. The photocurrent increases with the electric field and begins to saturate after 10 V/μm. After the irradiation is terminated, the photocurrent rapidly drops to a dark current level, demonstrating almost negligible signal lag. A detailed analysis of the temporal performance (evaluated in terms of signal lag and ghosting) of the a-PbO-based detectors can be found in [20,21].

**Figure 3:**
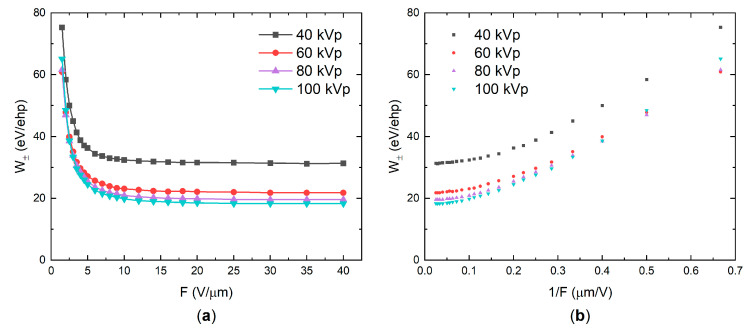
(**a**) $W_{\pm }$ as a function of the electric field at different X-ray tube voltages. *W*_±_ decreases with the field and the energy of X-rays. (**b**) The same values replotted as a function of the reciprocal field 1/*F*.

$W_{\pm }$ values were calculated using Equation (1) and plotted as a function of the applied electric field or the reciprocal electric field for different tube voltages in Figure 3a,b, respectively. For the reasons discussed later in the text, the tube current and source-to-detector distance (SDD) were adjusted for each tube voltage to keep a constant exposure level in the photoconductor’s plane of 100 mR. As is evident from Figure 3a,b, the sensitivity improves ($W_{\pm }$ decreases) as the field increases, rapidly saturating after 10 V/μm. The saturated values of $W_{\pm }$ depend on the tube voltage: 31, 22, 20, and 18 eV/ehp for 40, 60, 80, and 100 kVp, respectively. As it can be seen, $W_{\pm }$ decreases with increasing tube voltage, and thus with the mean energy of X-ray photons in the beam (see inset in Figure A1a).

**Figure 4:**
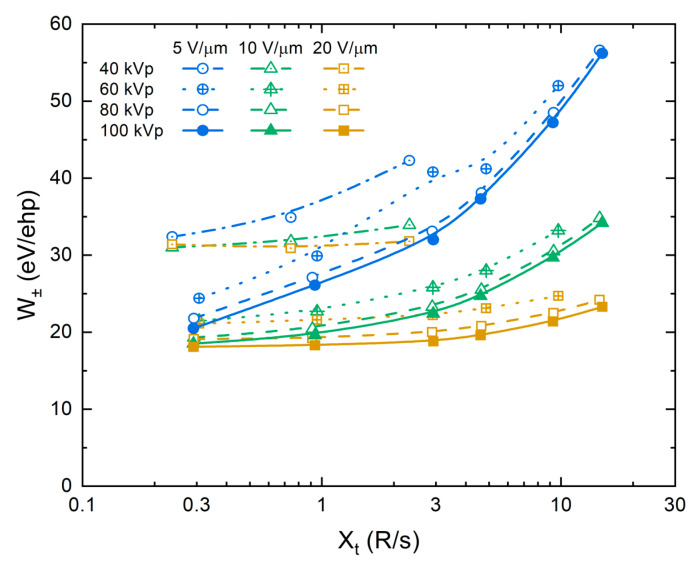
$W_{\pm }$ as a function of the exposure rate in different electric fields and at different tube voltages. $W_{\pm }$ increases with the exposure rate.

The effect of exposure (X-ray flux) is examined in Figure 4. It was found that $W_{\pm }$ changes with the exposure rate, but not with the exposure itself (i.e., $W_{\pm }$ is identical for two X-ray pulses with the same amplitude but different duration). Therefore, the exposure X dependency of $W_{\pm }$ was measured at a fixed pulse duration $t_{pulse}=0.1\;s$ and plotted as a function of exposure rate $X_{t}=X/t_{pulse}$. Figure 4 shows these results in different electric fields and at different tube voltages: $W_{\pm }$ increases with the exposure rate. At lower fields, $W_{\pm }$ changes more drastically: almost 200% growth when the exposure changes by a factor of 50. The rate of change is similar for different tube voltages. It should be noted that the exposure rates used were much larger than typical radiation levels used in the clinical practices (~10^−4^ R/s for fluoroscopy and ~10^−1^ R/s for 3D mammography [61,62]). However, it was not feasible to use exposures in the micro-roentgens range due to the limited sensitivity of the oscilloscope.

Since the exposure rate significantly affects the detector’s sensitivity, all experiments were performed with the exposure fixed at the lower end of the available range (100 mR per 0.1 s), unless otherwise is specified. This was achieved by adjusting the tube current and SDD.

To investigate the effect of the X-ray photon energy on the $W_{\pm }$, one has to use a measure of energy that would take the complex shape of a polyenergetic X-ray spectrum into account. The most common parameters are the tube voltage kVp (or, equivalently, the maximum energy of X-ray photons in a beam) and the mean energy $E_{mean}$ (calculated as the energy-weighted average). However, it should be noted that neither of these parameters characterizes a polyenergetic spectrum unambiguously and thus they should be treated as an approximate measure of the beam energy only [63].

**Figure 5:**
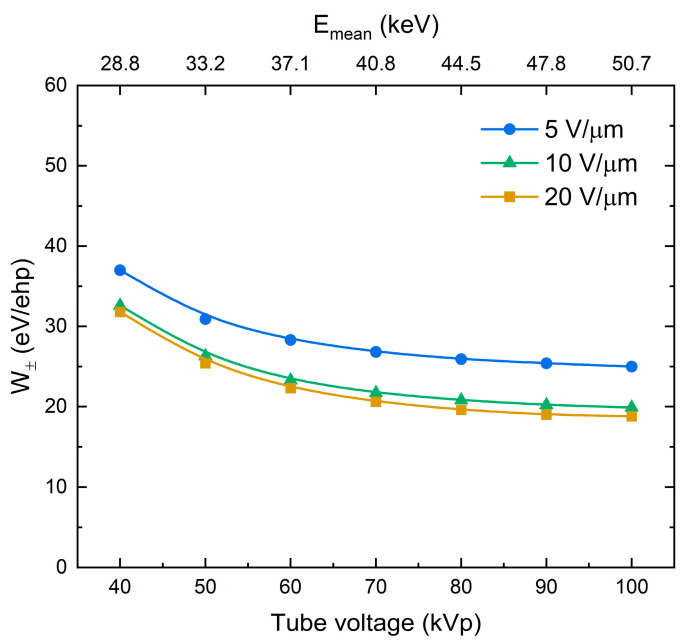
$W_{\pm }$ as a function of tube voltage at different electric fields. $W_{\pm }$ decreases as the energy of X-rays increases.

$W_{\pm }$ for different X-ray tube voltages and corresponding mean X-ray energies are shown in Figure 5. The detector’s sensitivity improves ($W_{\pm }$ decreases) as the energy of X-rays increases.

**Figure 6:**
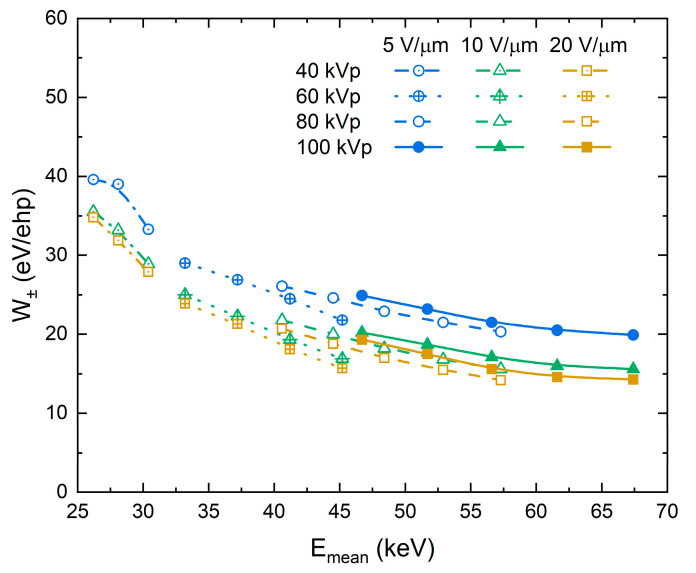
$W_{\pm }$as a function of the mean energy of X-ray photons at different tube voltages and electric field strengths. $W_{\pm }$ decreases as the energy of X-rays increases.

An alternative way to vary photon energy is by hardening the X-ray beam with added Al filtration. At a fixed tube voltage, a thicker Al filter attenuates the low-energy end of the spectrum and effectively shifts the mean energy towards a higher value. Figure 6 shows $W_{\pm }$ values as a function of mean X-ray energy for different electric fields and tube voltages. Within each tube voltage group, $W_{\pm }$ decreases as the mean energy increases. A discrepancy between $W_{\pm }$ values at the same *F* and $E_{mean}$, but different tube voltage, is not surprising, since, as it was mentioned earlier, $E_{mean}$ alone is not a sufficient parameter to characterize the incident polyenergetic X-ray beam. Nevertheless, the trends of the dependencies in Figure 5 and Figure 6 closely resemble each other.

**Figure 7:**
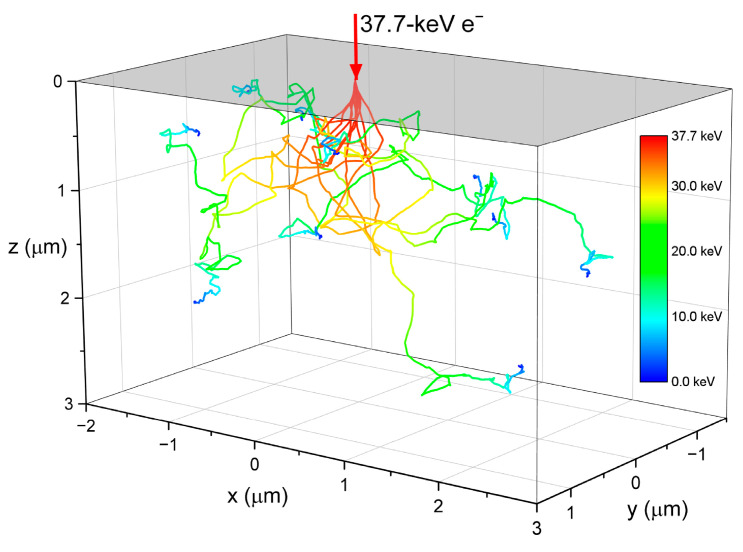
Typical electron trajectories in PbO for a 37.7-keV electron beam.

The electron transport was simulated using the Monte Carlo software CASINO [57]. Table 1 summarizes the results of the simulations and Figure 7 shows an example of typical electron trajectories for 37.7-keV incident electrons in the PbO sample. The electron beam energy of 37.7 keV represents the kinetic energy of a primary photoelectron ejected from the *L_3_*-subshell with the binding energy of 13 keV by the 100 kVp X-ray beam with a mean energy of 50.7 keV. The sample was irradiated by an electron beam from the top side; the electron trajectories are coloured according to their kinetic energy. As the primary electron traveled through the photoconductor, it collided with the atoms and gradually lost its energy. The average energy dissipated in a collision event did not appreciably vary with the initial energy of the primary photoelectron (Table 1). However, the average distance between the collisions (i.e., mean free path) and total path length (i.e., electron range) increased with the primary photoelectron energy, resulting in a declining energy deposition rate (Table 1).

## 5. Discussion

The obtained experimental results show well-pronounced dependencies of $W_{\pm }\;$on the electric field, X-ray energy, and exposure rate. Now we will try to take into account the presented dependencies in the recombination models, as was previously done for a-Se [33,34,36].

Firstly, let us consider a field dependency in a-PbO demonstrated in Figure 3. $W_{\pm }\left(F\right)$ firstly rapidly decreased according to 1/F (in the range of fields 1–10 V/μm), but started to saturate at higher fields with no further improvement observed, as is seen in Figure 3b. Replotting results from Figure 3 as η(F) yields $R_{SI}=0.6-2\;\mu m/V$, depending on the X-ray energy; however, for a-PbO with $\varepsilon_{r}=26$, Onsager theory requires a value of $R_{SI}=0.041\;\mu m/V$, which plays against the geminate recombination model as a plausible mechanism for photogenerated charge carrier loss in a-PbO.

Furthermore, analysis of the energy deposition during ehps generation and the X-ray energy dependency of the recombination rates rule out the geminate model completely. Indeed, Table 1 shows that the average portion of energy dissipated in a scattering event almost did not change with X-ray energy. Since photoelectric absorption is the main photon interaction mechanism in PbO for the diagnostic X-ray energy range [64], the deposited energy primarily causes ionization and excitation of atoms, i.e., ehps generation. Therefore, the same amount of ehps, on average, is generated in each collision event, and the separation of the geminate pairs remains the same. If geminate recombination is a dominant process, $W_{\pm }$ will be independent of X-ray energy. However, this is not the case: Figure 5 and Figure 6 clearly illustrate that $W_{\pm }$ monotonically decreases with the energy of X-rays. This behaviour disagrees with Onsager formalism but adheres to the columnar model. The decrease of $W_{\pm }$ with gradually increasing energy of X-rays is due to the reduction in the columnar recombination rate caused by the lowering of the photogenerated charge carrier density along the track of primary photoelectron (since the average distance between ionizing events increases, as was demonstrated by our Monte Carlo simulations (Table 1)). Therefore, geminate recombination can be excluded from the reasons for carrier loss in a-PbO, leaving columnar recombination as the dominant process.

**Figure 8:**
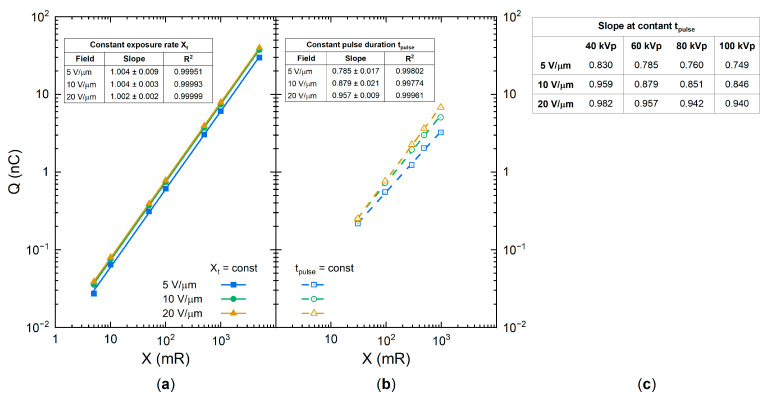
Charge collected versus the exposure for (**a**) the constant exposure rate and (**b**) the constant pulse duration at 60 kVp irradiation. (**c**) Slope values for the case of the constant pulse duration at different kVp and field strengths.

Let us now examine the exposure dependency of the collected charge and $W_{\pm }$. For this, the collected photogenerated charge was measured at a constant exposure rate and plotted in Figure 8a as a function of exposure in a log–log scale. The collected charge increased strictly linearly with the exposure, as demonstrated by the unity slope values in the inset to Figure 8a. In this case, both the number of collected ehps and the total energy absorbed were proportional to the exposure, and therefore, $W_{\pm }$ remained unchanged (see Equations (1), (A2) and (A4)). However, if the collected charge is measured at a variable exposure rate and a fixed pulse duration $t_{pulse}$, its dependency on the exposure is different. This is shown in Figure 8b: the slope values deviate from unity, and thus $W_{\pm }$ changes, as was demonstrated in Figure 4. At the lower field of 5 V/μm, the collected charge increased as $Q\sim X^{\alpha }$ with an intermediate exponent α=0.785; and at the higher field of 20 V/μm, it changed almost linearly: α=0.957 (see slope values in the inset to Figure 8b). In addition, the slope value decreased with increasing X-ray energy (Figure 8c). Since the exponents take an intermediate value between that for the bulk recombination (α=0.5) and columnar recombination (α=1), this analysis suggests the interplay between bimolecular Langevin recombination in the bulk and the column. Indeed, the carriers first experience the initial columnar recombination, and afterward, the escaped carriers drift through the bulk of the photoconductor and recombine with the carriers from the different columns, giving rise to the bulk Langevin recombination.

Although the above considerations allow for a qualitative model of X-ray generation and recombination in a-PbO, the saturation of $W_{\pm }$ at energy-dependent values well above the intrinsic $W_{\pm }^{0}$ remains unclear. The lowest experimentally achievable $W_{\pm }\;$ranges from 18 eV/ehp at 100 kVp to 31 eV/ehp at 40 kVp (to be compared with energy-independent $W_{\pm }^{0}$ around 5–6 eV/ehp, as suggested by Klein and Que–Rowlands rules for lead oxide with $E_{g}=1.9\;\mathrm{eV}$ [1]).

The saturation of $W_{\pm }$ has been previously observed in the High-gain Avalanche Rushing Photoconductor (HARP) detector with a-Se photoconductor at high electric fields [36]. Indeed, $W_{\pm }$ in a-Se initially decreases with the field as 1/*F*. However, in the fields stronger than 80 V/μm, $W_{\pm }$ saturated at a level of ~9 eV/ehp. This saturated value is larger than that theoretically predicted by Klein rule, 5–7 eV/ehp. Such behaviour is explained by the modified columnar recombination model which takes into account that the recombination rate is limited by the smaller of two parameters: time needed for carriers to meet in space and duration of the recombination event itself. In a high electric field, the time for an electron and hole to meet in space becomes smaller than the time needed for the recombination of the electron–hole pair that is on a scale of ~10^−12^ s. As the result, charge drift no longer influences the probability of recombination, which becomes independent of the electric field, and $W_{\pm }$ saturates.

Although saturation of $W_{\pm }$ in a-PbO occurs in an electric field weaker than that for a-Se, it confirms the findings in [36] that the Langevin recombination mechanism should not be expected at strong electric fields. As is shown in Table 1, the energy-dependent mean free pass $r_{MFP}\;$between the ionizing collisions in a-PbO is at a scale of several nanometers. This distance can be approximated as a maximum separation between the oppositely charged non-geminate carriers $r_{0}$ (although, realistically, $r_{MFP}$ significantly overestimates a mean separation $r_{0}$, taking into account that at this distance not a single ehp is created, but rather multiple ehps that form a dense electron cloud—a spur (see Appendix B)). Considering the intrinsic $W_{\pm }^{0}\approx \;$5 eV/ehp for a-PbO, the number of pairs generated in each spur could be estimated from the dissipated energy per collision (~35 eV, see Table 1) as ~7 ehps per spur, providing $r_{0}\approx \;$10^−7^ cm. Now, assuming that the mobility of holes (faster carriers in PbO) μ≈ 1 cm^2^/(V·s) at F=10 V/μm where $W_{\pm }\left(F\right)$ saturation begins (which seems a reasonable assumption for hole mobility in PbO at 10 V/μm [65]), the hole drift velocity $v_{d}=\mu F\approx \;$10^5^ cm/s. Therefore, the time $\tau =r_{0}/v_{d}$ that defines the probability for recombining carriers to meet in space is in the order of 10^−12^ s—shorter than the characteristic time of the recombination event for carriers of opposite sign placed at the same spatial point [36]. Similarly to a-Se, in strong electric fields the recombination in a-PbO becomes limited by the duration of the recombination event: the recombination rate no longer depends on the electric field. Therefore, $W_{\pm }$ saturates, as was demonstrated in Figure 3. This also explains the saturation of $W_{\pm }$ at different values depending on the X-ray energy. As the mean X-ray energy in a beam increases from 28.8 to 50.7 keV, the mean free path of the primary photoelectron $r_{MFP}$ increases by a factor of 1.7 (Table 1). This results in a reduced initial recombination rate and a saturated $W_{\pm }$ that is lower by the same factor (Figure 3).

## 6. Conclusions

The X-ray sensitivity in terms of the electron–hole pair creation energy $W_{\pm }$ of a single-pixel PI/a-PbO direct conversion X-ray detector prototype was characterized in a wide range of electric fields, X-ray photon energies (in diagnostic energy range), and exposures using polyenergetic irradiation. $W_{\pm }$ decreased with electric field strength, and above 10 V/μm saturated at 18–31 eV/ehp, depending on the energy of the X-rays—higher photon energy resulted in a lower $W_{\pm }$. In addition, $W_{\pm }$ increased with radiation exposure rate, especially in weaker electric fields. This demonstrates that the PI/a-PbO detector performs best in strong, practical electric fields (10–20 V/μm) in the diagnostic energy range and under low exposures, offering improved sensitivity as compared to a-Se.

The analysis of the field, X-ray energy, and exposure dependencies of the $W_{\pm }$ indicated an interplay between Langevin recombination within the ionization column (i.e., columnar recombination) and bulk Langevin recombination, which together are responsible for the carrier loss and suboptimal $W_{\pm }$ in a-PbO in electric fields weaker than 10 V/μm. In stronger fields, the columnar Langevin recombination cannot account for the observed field dependency of $W_{\pm }$, as the recombination process is no longer determined by the probability of X-ray-generated carriers meeting in space.

## Figures and Tables

**Table 1:** Results of the CASINO simulations.

Tube Voltage (kVp)/ Mean X-ray Energy (keV)	Electron Energy (keV)	Dissipated Energy Per Collision (eV)	Mean Free Path (nm)	Range (μm)	Energy Deposition Rate (eV/nm)
40/28.8	15.8	35.3	3.9	1.6	11.8
60/37.1	24.1	33.9	5.0	3.3	9.0
80/44.5	31.5	33.4	5.9	5.1	7.7
100/50.7	37.7	33.6	6.5	6.8	7.0

**Figure A2:**
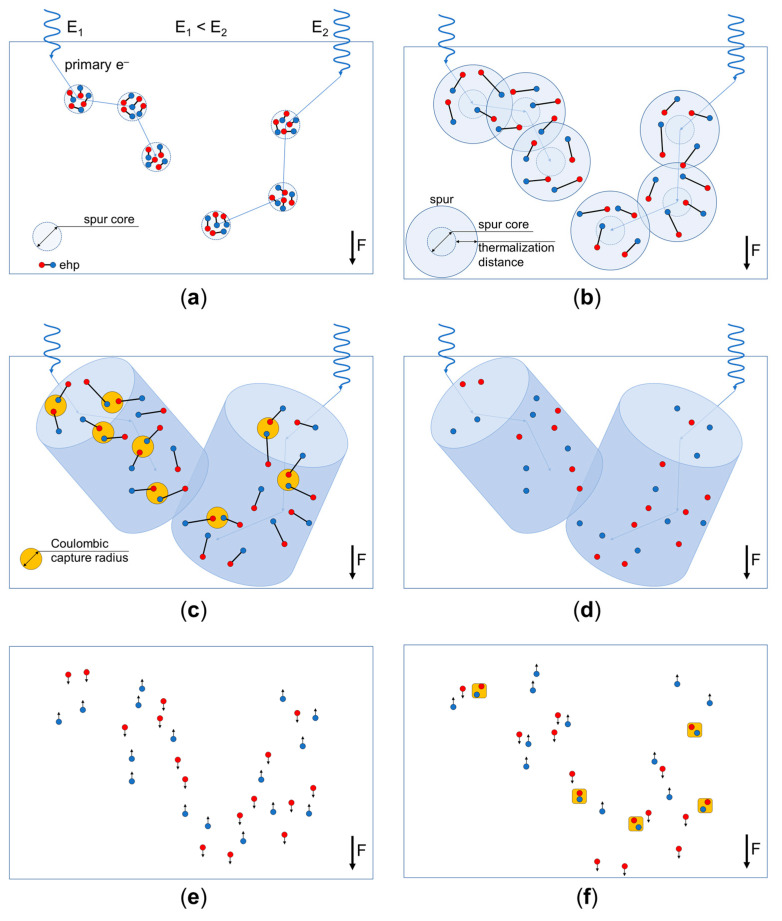
Schematic illustration of the charge generation and recombination processes. (**a**) A primary photoelectron ejected by an incident X-ray photon creates multiple ehps in each ionization event. (**b**) Secondary charge carriers thermalize in a drift-diffusion process and form a spur. (**c**) Overlapping spurs produce an ionization column along the track of the primary photoelectron. Oppositely charged carriers with a separation smaller than the Coulombic capture radius recombine. (**d**) The remaining carriers escape columnar recombination and (**e**) drift through the bulk of the photoconductor. (**f**) Drifting carriers from different columns and spurs recombine in the bulk.

## Data Availability

The data presented in this study are available on request from the corresponding author.

## Appendix A. X-ray Sensitivity Calculations

In this work, the detector was irradiated with a polyenergetic X-ray beam. Therefore, to accurately calculate the absorbed energy, the shape of the X-ray spectrum must be considered. The X-ray spectrum for the tungsten target at a given tube voltage, tube current, Al filtration, and source-to-detector distance (SDD) was simulated using the standard Tucker–Barnes–Chakraborty (TBC) model [66].

The validity of the model was verified by a half-value layer (HVL) of Al. Inherent filtration (glass, oil, and Al) can be adjusted in the model until a close match between the experimental and simulated HVL values is achieved, indicating that the modeled spectrum closely represents the one generated by the X-ray unit. To derive an experimental HVL of the beam, the exposure was measured for a naked tube and with added Al filtration of a different thickness up to 3 mm. The data were then interpolated to determine the Al thickness required to reduce the exposure to half of its original value.

The simulated spectrum represents the X-ray photon fluence incident on the detector (the number of photons for each energy $E_{i}$, per unit area per unit exposure) $N\left(E_{i}\right)$. The fraction of photons absorbed in the photoconductor is given by the energy-dependent Beer–Lambert law [63]:

(A1) $$N_{abs}\left(E_{i}\right)=N\left(E_{i}\right)\cdot \left(1-\exp\left\{-\left(\frac{\mu \left(E_{i}\right)}{\rho }\right)\cdot \rho \cdot d\right\}\right),$$

where (μ(Ei)ρ) is the mass attenuation coefficient; *ρ*—density; *d*—thickness of a-PbO layer. The absorbed energy is then calculated by summing the absorbed fraction (=(μen(Ei)ρ)(μ(Ei)ρ)) of the energy fluence ($=N\left(E_{i}\right)\times E_{i}$) over the entire energy range. For the detector with an area *A* and an incident exposure *X*, the total absorbed energy is:

(A2) Eabs=A·X·∑i((μen(Ei)ρ)(μ(Ei)ρ)·Nabs(Ei)·Ei),

where (μen(Ei)ρ) is the mass-energy absorption coefficient. The mass attenuation and mass-energy absorption coefficients for PbO were derived from the elemental coefficients for Pb and O (obtained from the NIST database [58]) as averages weighted by atomic mass.

The effective area of a detector is determined by the area of a smaller electrode, which in our case was a top gold contact. The exposure $X^{\prime }$ was measured with an ionization chamber located at a distance $d_{1}$ from the tube’s anode, away from the detector at a distance $d_{2}$. The exposure in the detector’s plane was then calculated by the inverse square law. Since the photoconductor was irradiated through the 0.7 mm thick glass substrate, its attenuation was also considered. The transmittance of the substrate $T_{s}$ was separately measured for each spectrum as the ratio of exposures with and without a "blank" substrate (i.e., the glass substrate without the photoconductor film deposited) in front of the X-ray tube window with all other parameters fixed. Finally, the incident exposure on the photoconductor is therefore given as:

(A3) $$X=X^{\prime }\cdot {\left(\frac{d_{1}}{d_{1}+d_{2}}\right)}^{2}\cdot T_{s}\;.$$

The number of collected photogenerated ehps was obtained from the current transients. The dark current was subtracted from the current transient, and the resulting photocurrent was integrated over the pulse duration. The amount of charge was divided by an elementary charge *e* to obtain the number of carriers collected:

(A4) Nehp=∫Iphoto(t)dte .

The simulated X-ray spectra for the X-ray tube with a tungsten target and 2 mm added Al filtration at different tube voltages are shown in Figure A1a. The low-energy end (up to ~15 keV) was attenuated by the inherent filtration (glass housing) and added Al filtration. The peaks at 58–59 keV and 67–69 keV are due to the emissions of the characteristic $K_{\alpha }$ and $K_{\beta }$ X-ray photons of tungsten, respectively. The spectra were normalized to 1 R of incident exposure for better representation. The inset shows the mean energy, HVL, and exposure at a given tube voltage and typical parameters (2 mm added Al filtration, tube current 200 mA, pulse duration 0.1 s, SDD 80 cm) for unnormalized X-ray beams.

Figure A1b shows measured exposure as a function of the added Al filtration thickness to the naked tube for selected tube voltages. The calculated and measured HVL values for the naked tube are listed in the inset: the difference is <2% for all tube voltages. The error of 2% in HVL value translates into 1% uncertainty in the calculated value of absorbed energy $E_{abs}$, and therefore, of $W_{\pm }$, which is smaller than a symbol size in Figure 3, Figure 4, Figure 5 and Figure 6.

## Appendix B. Model for the Charge Generation and Recombination Processes in a-PbO

A qualitative model of the charge generation and recombination processes in a-PbO is based on our experimental results, and previous experimental observations and theoretical simulations in a-Se.

Upon impinging on the photoconductor, the energy of the X-ray photon (minus the binding energy of the electron) is mostly transferred to the kinetic energy of a primary electron, since the photoelectric absorption is the main photon interaction mechanism in PbO for the diagnostic X-ray energy range [64]. The kinetic energy of the primary photoelectron is deposited into the material during the inelastic collisions with the outer-shell atomic electrons, which leads to the ionization of these atoms (creation of the ehps) or emission of a phonon (energy loss). A single ionization event can result in the creation of multiple ehps in the vicinity of the interaction site, composing a spur core (Figure A2a). This event could be interpreted as the excitation of plasma waves, which very quickly decay into multiple ehps [53]. After the ehps are created, they diffuse away from the excitation location in a thermalization process and gradually lose their initial kinetic energy. At the end of the thermalization process, ehps are separated by a finite thermalization distance (which can be estimated by the Knights–Davis equation [67], but usually is interpreted as a free fitting parameter), constituting an electron cloud—namely, a spur (Figure A2b). As the primary electron makes its way through the photoconductor, it collides with the atoms and creates many localized spurs along its track. If the ionization density along the track is large enough, individual spurs overlap and form a column of X-ray-generated secondary ehps surrounding the electron’s track [41,53] (Figure A2c).

If at the end of the thermalization process, the distance between any oppositely charged carriers is smaller than the Coulombic capture radius (such that their mutual attraction is stronger than the thermal diffusion and the drift in the applied electric field), then the carriers will recombine (Figure A2c). Due to a high density of the X-ray-generated carriers inside the column [32,34,41,53], the mean separation of the twin ehp is larger than the separation between any adjacent non-geminate electrons and holes, meaning that non-geminate ehps are more likely to recombine than the geminate pairs, leading to a columnar recombination mechanism. The carriers with separation larger than the Coulombic capture radius are likely to escape the recombination (Figure A2d) and contribute to the X-ray signal, although the probability of escape depends on the combined effects of the diffusion and extraction fields [34,44,67]. A fraction of the electrons and holes that escaped recombination will drift in the applied electric field towards the opposing electrodes where they are collected (Figure A2e). If columns are generated closely in space, the carriers from different columns and spurs can meet during their drift and recombine in the bulk (Figure A2f), contributing to the bulk Langevin recombination.

Photogenerated charge density is an important parameter in the columnar recombination model since it directly affects the recombination rate. It can be described in terms of the energy deposition rate. The rate of energy deposition by a primary electron (i.e., stopping power) decreases with its kinetics energy and so does with photon energy (see Table 1 and [68]). Thus, the density of ehps in the column decreases with increasing photon energy and ehps have a greater probability of escape [34,35,40,41,54]. Therefore, with increasing energy of the incident X-ray photon, the columnar recombination rate within the photoconductor decreases, increasing the fraction of charge collected.
